# Assessment of Lipophilicity Indices Derived from Retention Behavior of Antioxidant Compounds in RP-HPLC

**DOI:** 10.3390/molecules22040550

**Published:** 2017-03-29

**Authors:** Ioana Anamaria Sima, Agata Kot-Wasik, Andrzej Wasik, Jacek Namieśnik, Costel Sârbu

**Affiliations:** 1 Faculty of Chemistry and Chemical Engineering, Babeş-Bolyai University, Arany Janos Str., No. 11, RO-400028 Cluj-Napoca, România; ioanatuhutiu@ymail.com (I.A.S.); csarbu@chem.ubbcluj.ro (C.S.); 2 Faculty of Chemistry, Gdansk University of Technology, 80-233 Gdansk, Poland; agawasik@pg.gda.pl (A.K.-W.); chemanal@pg.gda.pl (J.N.)

**Keywords:** antioxidants, lipophilicity, reversed phase high-pressure liquid chromatography (RP-HPLC), PCA, sum of ranking differences

## Abstract

Reverse phase high pressure liquid chromatography was employed in order to evaluate the lipophilicity of antioxidant compounds from different classes, such as phenolic acids, flavanones, flavanols, flavones, anthocyanins, stilbenes, xantonoids, and proanthocyanidins. The retention time of each compound was measured using five different HPLC columns: RP18 (LiChroCART, Purosphere RP-18e), C8 (Zorbax, Eclipse XDBC8), C16-Amide (Discovery RP-Amide C16), CN100 (Saulentechnik, Lichrosphere), and pentafluorophenyl (Phenomenex, Kinetex PFP), and the mobile phase consisted of methanol and water (0.1% formic acid) in different proportions. The measurements were conducted at two different column temperatures, room temperature (22 °C) and, in order to mimic the environment from the human body, 37 °C. Furthermore, principal component analysis (PCA) was used to obtain new lipophilicity indices and holistic lipophilicity charts. Additionally, highly representative depictions of the chromatographic behavior of the investigated compounds and stationary phases at different temperatures were obtained using two new chemometric approaches, namely two-way joining cluster analysis and sum of ranking differences.

## 1. Introduction

A powerful weapon in the fight against free radicals consists of the polyphenolic compounds which exert significant biological resistance against oxidants. Research is suggesting that both the classical antioxidant properties of the polyphenolic compounds (given by the hydrogen-donating capacity through their molecular structure) and their metal-chelating properties (effectively preventing transition metals from catalyzing oxidation reactions) may be important elements in the overall effectiveness of such compounds against free radical oxidations [1].

In order to benefit from the properties of the polyphenolic compounds, it would be of great interest to conduct quantitative structure–property relationship (QSPR) studies regarding their lipophilicity. This parameter represents the extent to which a substance prefers a hydrophilic or a lipophilic medium and the distribution between media with different polarities suggests the behavior of a compound in experimental, natural, or biologic environments. Thus, in the human body, a compound with a high lipophilicity index (hydrophobic) will be distributed mainly in lipid bilayers and those with a low lipophilicity index (hydrophilic) will be distributed mainly in blood and serum. Knowing that this property of a molecule is particularly useful when considering the administration of any kind of medication, because lipophilicity determines several parameters of drugs, such as the ability to reach its target, the affinity for the target, and how long it will remain active in the body.

The methods for determining lipophilicity were classified by Sangster [2] in two categories: direct methods, in which the compound is quantitatively determined in one or both phases, and indirect methods, which do not require a quantitative analysis. The most popular direct method is the “shakeflask” method, whereas the chromatographic techniques are recognized as indirect methods for the determination of lipophilicity.

Generally, the chromatographic methods are based on determining the retention parameters and the most used method is the reversed phase high-pressure liquid chromatography (RP-HPLC) [3,4]. The principles of determination were established by Snyder and Kirkland [5], and in HPLC the affinity of a solute for the stationary phase is characterized by the retention factor (*k*), defined as *k = (t*_R_ *− t*_0_*)/t*_0_, where *t*_R_ is the retention time and *t*_0_ is the dead time.

It has been demonstrated experimentally that *logk* is linearly inter-correlated with the volume fraction of the organic co-solvent (*φ*, C), following the classic model of Soczewinski’s and Snyder’s equation [6]: *logk = logk_w_ − Sφ*, where *logk_w_* is considered to be the chromatographic lipophilicity index and represents the retention factor for pure water as eluent, *S* is widely associated with the solvent strength or with the sorbent specific surface area, and *φ* is the volume fraction of the organic modifier.

The direct measurement of *logk_w_* is the often very difficult, if not impossible, due to the fact that it can lead to very long retention time and at the same time to excessive broadening of the peak. For this reason, measuring k with different ratios of water-organic solvent mixture as mobile phases is preferred, and the extrapolation of the correlation between *logk* vs. % *organic modifier* indicates the value of *logk* when using only water as the mobile phase.

An alternative to the lipophilicity index is the so-called chromatographic hydrophobicity index or Valko’s index *φ*_0_ [7,8], which represents the volume fraction of the organic solvent in the mobile phase for which the amount of solute in the mobile phase is equal to that in the stationary phase (*k* = 1, *logk* = 0). This parameter is described using the equation: *φ*_0_ *= logk_w_/S*. Furthermore, in the final years, new lipophilicity indices were obtained using principal component analysis (PCA). Thus, the score corresponding to the first principal component (PC1), obtained by applying PCA to the matrix formed by the retention parameters (*k* and *logk*), has proven to give highly valuable information regarding the lipophilicity [3,4,9,10,11,12].

In addition, the continuous requirement of the pharmaceutical industry to have efficient methods to rapidly assess the lipophilicity of newly synthetized compounds, has made the use of computational approaches for the prediction of this parameter more popular. Their advantages arise out of the fact that they do not require any experimental work, which drastically reduces the costs. Thus, much computer software that calculates several lipophilicity descriptors estimated by different algorithms based on structural, topological, or property considerations has been developed [13]. Mannhold et al. [14] present, in their review, the state-of-the-art in the development of logP prediction approaches and detailed description of the methodology background of the major categories: substructure-based methods (fragmental, atom-based) and property-based methods (empirical approaches, 3D structure based, topological descriptors) are also given.

Furthermore, in order to compare, classify, and determine the best experimental method or computational approach for the determination of lipophilicity, a novel method of assessment has recently been developed by K. Heberger, namely the sum of ranking differences (SRD) [15]. This methodology has been successfully applied for the comparison of calculated lipohilicity scales with indices derived from retention behavior on RP-HPLC and HPLC–hydrophilic interaction columns [16]; the comparison of lipophilicity measures obtained by typical RP-TLC experiments with in silico approaches and lipophilicity measures obtained by micellar chromatography and typical RP-TLC experiments combined with in silico approaches [17]; liphophilicity measures comparison with classical and novel chemometric methods [18].

In view of the above considerations, the aim of this study was to assess the lipophilicity indices derived from the retention behavior of antioxidant compounds, estimated on five different HPLC columns at two different temperatures, as well as to compare the experimental indices with those obtained through computational approaches.

## 2. Materials and Methods

The investigated compounds in this study belong to several groups of antioxidants, which are as follows: *phenolic acids*: (**1**) *p*-coumaric acid, (**2**) ferulic acid, (**5**) protocatechuic acid, (**6**) sinapic acid, (**7**) caffeic acid, (**8**) vanillic acid, (**11**) chlorogenic acid, and (**17**) gentisic acid; *flavonoids: flavanones*: (**3**) naringenin, (**4**) naringin, (**13**) hesperetin, and (**14**) hesperidin, *flavanols:* (**9**) (+)-catechin, (**10**) (−)-epicatechin, and (**15**) (−)-epigallocatechin gallate, *flavonols*: (**12**) rutin, and *flavones*: (**18**) apigenin; *anthocyanins*: (**16**) pelargonidin; stilbenes: (**19**) pterostilbene and (**21**) resveratrol; *xantonoids*: (**20**) mangiferin; *proanthocyanidins*: (**22**) C1 type proanthocyanidin. For each compound, solutions in concentration of 1 mg/mL in methanol were prepared using HPLC grade standards obtained from commercial sources (Merck, Kenilworth, NJ, USA; Fluka, St. Louis, MO, USA).

The chromatographic measurements were performed on an Agilent 1100 Series LC system consisting of a vacuum degassing unit, a binary high pressure pump, a standard automatic sample injector, a column thermostat and a diode array detector (DAD). In order to obtain the lipophilicity indices, the retention time of each compound was measured using five different HPLC columns: RP18 (LiChroCART, Purosphere RP-18e, 3 mm × 125 mm, 5 µm particle size, carbon load—18%), C8 (Zorbax, Eclipse XDBC8, 4.6 mm × 150 mm, 5 µm particle size, carbon load—7.6%), C16-Amide (Discovery RP-Amide C16, 4.6 mm × 150 mm, 5 µm particle size, carbon load—11%), CN100 (Saulentechnik, Lichrosphere, 4 mm × 250 mm, 5 µm particle size), and pentafluorophenyl (Phenomenex, Kinetex PFP, 2.1 mm × 150 mm, 2.6 µm particle size). The mobile phase consisted of a mixture of methanol and water (0.1% formic acid) in different proportions and the injection volume was 1 µL of standard solution (1 mg/mL).

The retention times were measured at 22 °C and 37 °C by the UV detector at 254 nm and for each solute, the retention factor expressed as *k = (t_r_* − *t*_0_*)/t*_0_, was determined at different proportions of methanol. Then, a plot was made using *logk* vs. % *methanol* (in mobile phase), and the extrapolation to 0% methanol gave *logk_w_*. The dead time was measured for all selected columns using urea and they were as follows: *t*_0_ (RP18) = 0.903 min, *t*_0_ (C8) = 1.614 min, *t*_0_ (C16-Amide) = 1.275 min, *t*_0_ (CN) = 2.135 min, and *t*_0_ (PFP) = 1.766 min. The measurements were carried out at a flow rate of 0.7 mL/min for RP18, C8, C16-Amide, CN columns and 0.2 mL/min for PFP column. In all cases, five different methanol fractions were used for the extrapolation to *logk_w_*.

Additionally, in order to compare the experimental results, some lipophilicity descriptors were calculated using different software, such as Chem 3D Ultra 8.0 (http://www.cambridgesoft.com), Dragon Plus version 5.4 (http://www.talete.mi.it), and ChemDoodle (https://www.chemdoodle.com). Thus, 12 descriptors were calculated as follows (Table S1): ChemDraw Ultra 8.0 provided five logP values (LogP, CLogP, LogP^C^-Crippen’s method, LogP^V^-Viswanadhan’s method, LogP^B^-Broto’s method) calculated on the basis of fragmental and atom based methods; another four logP values were calculated by the software Dragon 5.4 using topological descriptors (MLOGP-Moriguchi’s method, MLOGP2–Squared Moriguchi’s method, ALOGP–Ghose-Crippen’s method, and ALOGP2–Squared Ghose-Crippen’s method), and ChemDoodle provided three values (NCNHET, AlogP98, XLogP2).

Chemometrics analyses were performed using the Statistica 8.1 software (StatSoft, Tulsa, OA, USA), and CRRN_DNA_V6_S computer code (Excel extension) developed by K. Heberger et al. was used for ranking and classification of indices [15].

## 3. Results and Discussion

The group of antioxidants investigated in this study includes compounds with very different structures, sizes, and polarities, so it is expected that they have quite different chromatographic behavior. Therefore, the methanol fraction contained in the mobile phase was optimized so that all compounds have retention times between *t*_0_ (dead time) and a maximum of 15 min so that the analysis duration is as short as possible and that the results for different temperatures (22 °C and 37 °C) can be compared. Thus, the fraction of methanol, for which a linear range was obtained for *logk*, ranged between 50–60% for the RP18 and CN columns, 60–70% for the C8 and C16-Amide columns, and 55–65% for the PFP column; and in all cases an increment of 2.5% was used to obtain the five specified concentrations. The strong linear dependence of retention parameters through the methanol fraction variance was demonstrated by the values of determination coefficient (*R*^2^) higher than 0.99 in all cases.

Furthermore, by evaluating the profiles of k and *logk* values for all methanol fractions determined for both 22 °C and 37 °C, the regular changes in retention with increasing methanol ratios were observed in the case of C8, C16-Amide, PFP (except Compound **22**), and CN column, except RP18. In the case of the four columns, the mk and m*logk* parameters were overlapping the intermediate (median) value corresponding to the middle concentration of methanol (Figure S1a–e).

All the specific chromatographic lipophilicity parameters (arithmetic mean of k and *logk*- m*k* and m*logk*, *logkw*, *S*, *φ*_0_, scores corresponding to the first principal component obtained by applying PCA to the retention data—PC1/*k* and PC1/*logk*) were calculated and considered for all investigated columns at 22 °C and 37 °C, and the obtained results are presented in Tables S2 and S3. By summary evaluation, it can be observed that at 22 °C pterostilbene has the highest lipophilicity index for the C8, C16-Amide, and CN columns, pelargonidin for the RP18 column, and procyanidin C1 for the PFP column, while at 37 °C pterostilbene has the highest lipophilicity index for the RP18, C8, and C16-Amide columns, pelargonidin for the CN column, and apigenin for the PFP column. Additionally, the lowest lipophilicity index at 22 °C was found for epigallocatechin gallate on RP18 column, procyanidin C1 on C8 column, protocatechuic acid on C16-Amide and PFP columns, and chlorogenic acid on CN column, while at 37 °C the lowest lipophilicity index was found for catechin on RP18 and C16-Amide columns, and procyanidin C1 on C8, CN, and PFP columns.

In order to see how the temperature affects the lipophilicity, we will refer only to the indices *logk_w_* and m*logk*. First, matrices of correlation between the data obtained at 22 °C vs. 37 °C for all columns, including the computational lipophilicity values, were calculated, and the obtained results are presented in Tables S2 and S3. Accordingly, it can be observed, considering firstly experimental *logk_w_* values for the two temperatures, the higher correlations were obtained for C16 (r = 0.969), C8 (r = 0.983), and CN (r = 0.828). A low correlation was obtained for RP18 (r = 0.463), and surprisingly a very low negative value resulted for PFP (r = −0.042). The statistical results concerning the computational lipophilicity descriptors indicate that at 22 °C the highest correlation were obtained on PFP (r = 0.918 with NCNHET, r = 0.873 with XLogP, and r = 0.855 with ALogP2) and CN (r = 0.800 with CLogP and r = 0.620 with MLogP). On the other hand, at 37 °C the best correlations were obtained on CN column (r = 0.533 with ALogP98) and RP18 (r = 0.504 with CLogP). A high correlation was also found for RP18 column vs. Average value (r = 0.906) calculated for all experimental and computational data corresponding to each investigated compound; this value is used also in the Heberger algorithm [15,16,17,18], as will be discussed below. In addition, the results in Table S2 illustrate a significant correlation between the results obtained on all columns (with some exceptions in the case of PFP and RP18) and the following computational descriptors: CLogP, MLogP and Average.

**Figure 1:**
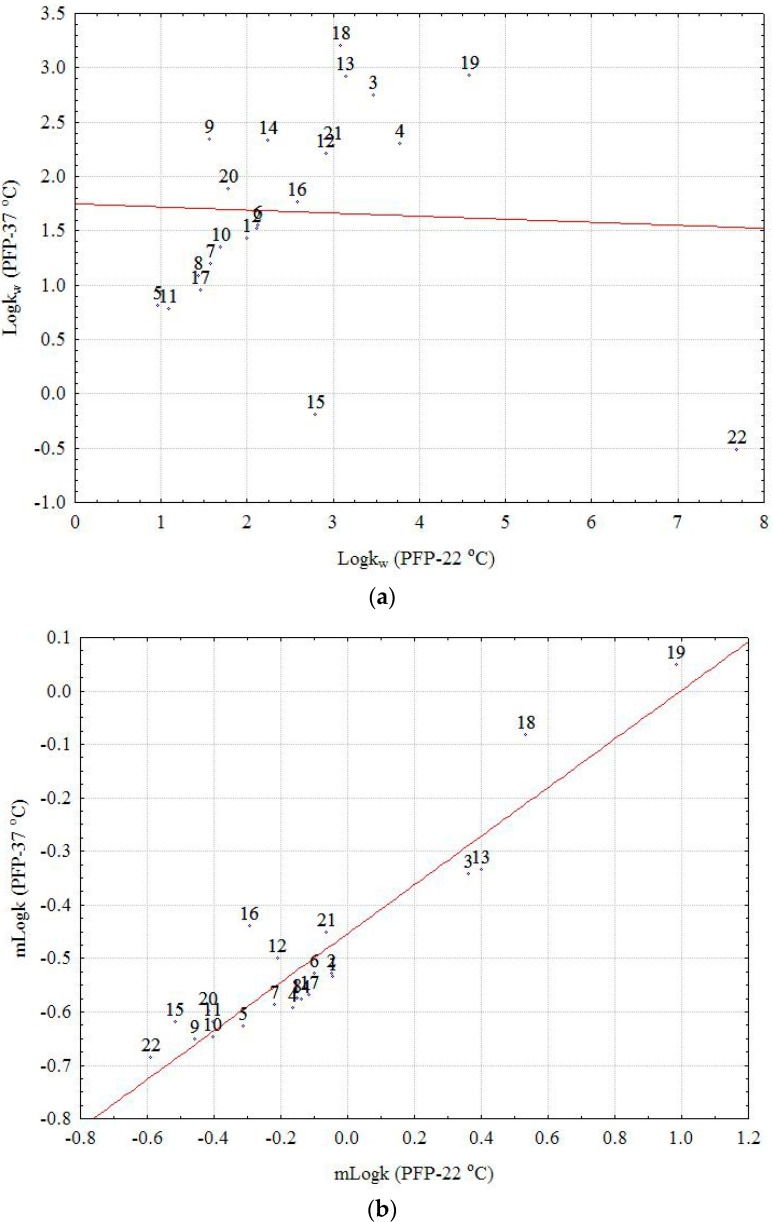
Scatterplot of *logkw* values corresponding to PFP column at 22 °C (**a**) and 37 °C; and m*logk*, respectively (**b**).

**Figure 2:**
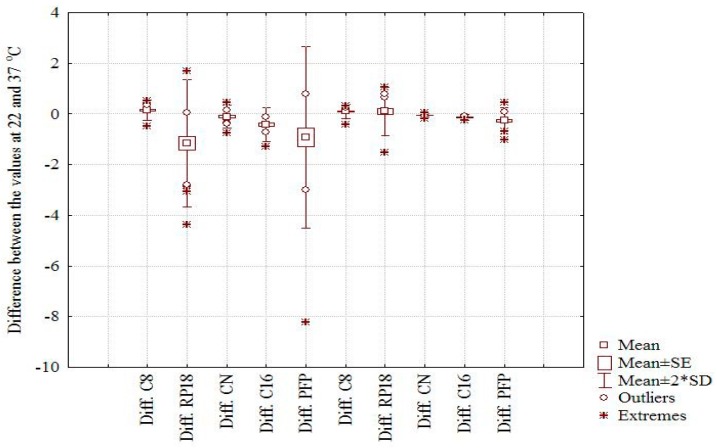
Box and whiskers corresponding to *logkw* values, (the first five boxes, from left to right) and m*logk* values, respectively (the last five boxes).

The statistical evaluation of the correlation results considering the experimental data estimated as m*logk* (Table S3) and the computationally indices showed that there is a high correlation between all experimental lipophilicity indices at the two temperatures excepting the correlations between RP18 and CN (22 and 37 °C; r = 0.342 and r = 0.239), PFP at 37 °C (r = 0.358), and C16 at 37 °C (r = 0.384). A significant correlation has been observed between the m*logk* values and CLogP (0.525 < r < 0.723), MLogP (0.423 < r < 0.679). A significant correlation is pointed out (with some exceptions) in the case of Average, ALogP98 and XLogP2. In addition, the correlation between m*logk* values at 22 °C and 37 °C for PFP becomes highly significant (r = 0.938). The large difference between the correlation coefficients obtained for *logk_w_* and m*logk* at the two temperatures in the case of PFP column can be clearly explained by the effect of extrapolation in the first case. The profiles of *logk_w_* and m*logk* presented in Figures S2a–e and S3a–e and the scatterplot of data corresponding to *logk_w_* and m*logk*, respectively, at the two temperatures, clearly illustrate a separate chromatographic behavior of Compound **22** (the outsized molecule with a big number of OH groups), which appears as a strong outlier (extreme) in the first case (Figure 1a,b). Moreover, the effect of temperature on the considered chemically bonded columns and the chromatographic behavior of the investigated compounds is clearly illustrated by the box and whisker plot depicted in Figure 2. The larger difference is observed in both cases on the RP18 and PFP columns and the small effect on C16 and CN, two columns with higher polarity. Considering the m*logk* values, a distinct difference can be seen between the nonpolar C8 and C18 columns (positive effect) and the CN, C16-Amide, and PFP (negative effect in the order CN < C16-Amide < PFP). The discrepancies observed in the case of *logk_w_* values can be explained once again by the effect of extrapolation and the different chromatographic behavior of certain compounds (**13**, **16**, **18**, **19**, and **22**).

The statements above are well supported by the results obtained applying classical hierarchical cluster analysis (HCA) and PCA on the standardized datasets. The dendrogram obtained in the case of dataset including experimental *logk_w_*, and computationally indices illustrate three well-separated clusters (Figure S4a). The *logk_w_* corresponding to CN and C16 columns at the two temperatures, including MLogP, are in the first group, the second combines the *logk_w_* obtained on C8 at the two temperatures, PFP and RP18 at 37 °C and some computational indices (ALogP, ALogP98, ClogP, and Average). The third cluster includes *logk_w_* corresponding to PFP and RP18 at 22 °C and XLogP2, NCNHET, MLogP2, and ALogP2. If the m*logk* values are considered, a clear distinction between computationally estimated logPs and chromatographic indices is obtained. The high similarity of the m*logk* is also clearly shown (Figure S4b).

Applying PCA on the *logk_w_* values, the first principal component explains 52.33% of the total variance, and the second component, 23.90%: a two-component model thus accounts for 76.23% of the total variance. The results from the PCA of m*logk* values are a little different. The first two PCs account for 75.58% of the total variance (PC1 54.24% and PC2 21.34%). The patterns obtained by two-dimensional representations of the loadings are more or less similar with the HCA-patterns discussed above. In the case of *logk_w_* (Figure S5a), two groups are clearly separated. The first include the majority of the experimental *logk_w_* indices and two computationally scales (ClogP and MLogP), in the second group appear two *logk_w_* (RP18-37 °C and PFP-22 °C) near the other computationally scales. Two major groups are present also in the case of the m*logk* dataset. The first group includes all the m*logk* indices and two computational scales (CLogP and MLogP), and in the second group we find only computationally scales (Figure S5b).

**Figure 3:**
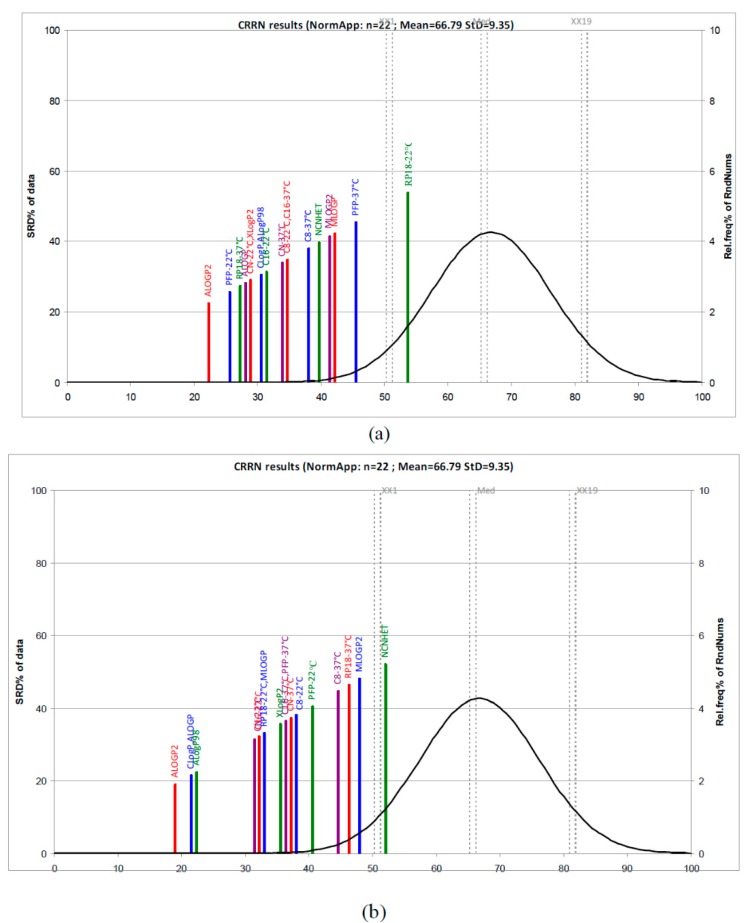
SRD-CRRN ranking of chromatographically estimated lipophilicity indices *logkw* (**a**) and m*logk* (**b**), and computationally calculated logP values.

At the same time, the lipophilic character similarities existing between the investigated compounds may be illustrated by the lipophilicity charts (“holistic lipophilicity chart”) obtained by 2-D scatterplots of the scores corresponding to the first two principal components. The score plots (Figure S6a,b) reveal two groups (more compacted in the case of *logk_w_*) and identify two outliers: pterostilbene (**19**) and C1 type proanthocyanidin (**22**). A two-way joining cluster analysis applied on a dataset formed by the *logk_w_* and m*logk* values obtained for all compounds on all investigated columns at the two temperatures including the computationally calculated indices provides similar conclusions regarding the effect of temperature and the chromatographic behavior of the compounds investigated (Figure S7a). The most similar results, considering *logk_w_* values and the computational scales, for example, are easily observed in the case of CN, C8, and C16 at the two temperatures (green color), and the outlier position of the C1 type proanthocyanidin (**22**) (yellow color) is also clearly indicated. The pattern in the case of m*logk* values including the computational scales illustrates a high similarity among all experimentally indices and CLogP, ALogP and Average appear to be closer to them (Figure S7b). In order to get more information and a better understanding of the experimentally and computationally estimation of lipophilicity, we also applied a new non-parametric ranking method, a sum of ranking differences-comparison of ranks by random numbers (SRD-CRRN) [15,16,17,18]. According to the SRD-CRRN, considering first the *logk_w_* values and computationally scales, the best descriptors are obtained using PFP-22 °C, RP18-37 °C, CN-22 °C, and C8-22 °C including ALogP2 (the best), ALogP, and CLogP. Lower ranking values were obtained in the case of RP18-22 °C, PFP-22 °C, and MLogP and MLogP2 (Figure 3). In the case of the dataset comprising m*logk* values and calculated LogP values, the results presented in Figure 3 indicate ALogP2, CLogP, and ALogP as the best computationally scales followed by two groups of lipophilicity measures: (CN, C16, and RP18 at 22 °C and MLogP) and (XLogP2, C16, and PFP at 37 °C, CN-37 °C, and C8-22 °C). The farthest group includes C8 and RP18 at 37 °C, as well as MLogP and NCNHET, and they are considered the worst lipophilicity measures.

## 4. Concluding Remarks

Investigations concerning the lipophilicity of a group of antioxidant compounds were conducted using reversed phase high-performance liquid chromatography. Different mixtures of methanol–water as mobile phase and several stationary phases, such as RP18, C8, C16-Amide, CN, and PFP were tested, and the results indicated pterostilbene as the most lipophilic compound. Significant correlations were obtained between different experimental indices of lipophilicity at the two temperatures and some computed logP scales (CLogP, MLogP, and ALogP98), and the mlogk values were the most correlated with the computed indices. In addition, the results obtained in this study by applying multivariate exploratory techniques, such as HCA, PCA, or the two-way joining clustering and profile representation, illustrated more or less the same (dis)similarities of the stationary phases and were well supported by the ranking scales generated applying SRD-CRRN algorithm. Overall, the results (mainly m*logk* indices) illustrate a similar and small effect of temperature on the chromatographic behavior of the investigated compounds in all cases. In consequence, we concluded that the mean (m*logk*) is a better lipophilicity estimator, as it is not affected as much by experimental and model errors like in the case of the extrapolation estimator (*logk_w_*), a conclusion which was also pointed out in the literature and well supported by these results.

## Supplementary Materials

Supplementary materials are available online.
