# Supplementary material for: Assessment of Lipophilicity Indices Derived from Retention Behavior of Antioxidant Compounds in RP-HPLC
Source: Molecules. 2017 Mar 29;22(4):550. doi: 10.3390/molecules22040550 (PMC6154650; doi:10.3390/molecules22040550)
Supplement: Supplementary file 1 [file molecules-22-00550-s001.pdf]

Table 1S. Log P values calculated by different computer software (ChemDraw Ultra 8.0, ChemDoodle and Dragon 5.4)

Compound	logP	logP[c]	logP[v]	logP[b]	CLogP	NC+NHET	ALogP98	XLogP2	MLOGP	MLOGP2	ALOGP	ALOGP2
1	1,572	1,540	1,870	1,570	1,572	2,210	0,948	1,925	1,429	2,042	1,635	2,673
2	1,416	1,420	1,620	1,700	1,421	2,120	0,956	2,132	1,193	1,422	1,618	2,619
3	1,629	1,630	1,990	1,130	2,445	2,560	2,185	3,756	1,450	2,101	2,298	5,279
4	-0,970	-1,100	0,090	-	-0,090	2,890	-1,489	3,051	-1,674	2,801	-0,456	0,217
5	0,445	0,810	1,180	0,450	1,062	1,790	0,796	0,979	0,592	0,351	0,900	0,809
6	1,290	1,290	1,360	1,830	1,204	2,120	0,965	2,339	0,964	0,930	1,602	2,566
7	1,153	1,150	1,580	1,180	0,975	2,010	0,653	1,251	0,889	0,791	1,367	1,870
8	0,965	1,080	1,210	0,970	1,355	1,900	1,099	1,860	0,926	0,857	1,151	1,324
9	1,504	1,500	2,100	0,800	0,534	2,450	1,222	2,270	0,264	0,061	1,920	3,687
10	1,504	1,500	2,100	0,800	0,534	2,450	1,222	2,270	0,264	0,061	1,920	3,687
11	-0,751	-0,750	0,020	-1,630	-1,879	2,230	-1,188	0,230	-0,663	0,439	-0,416	0,173
12	-2,277	-2,280	-0,860	-	-1,361	2,670	-1,687	0,965	-3,148	9,908	-1,446	2,090
13	1,503	1,500	1,730	1,260	2,294	2,560	2,194	3,963	0,927	0,859	2,281	5,204
14	-1,223	-1,220	-0,160	-	-0,291	2,890	-1,481	3,258	-2,176	4,735	-0,482	0,232
15	2,073	2,070	3,020	1,460	1,491	2,670	1,909	2,721	-1,317	1,736	2,894	8,375
16	-	-	-	-	2,161	2,560	3,203	1,950	1,253	1,570	3,088	9,538
17	0,445	0,810	1,180	0,450	1,622	1,790	0,796	0,533	1,103	1,217	0,900	0,809
18	1,904	1,900	2,320	1,870	2,905	2,560	2,577	2,750	1,764	3,112	2,334	5,449
19	3,911	3,580	3,470	3,910	4,175	2,890	2,929	5,200	2,908	8,454	3,516	12,362
20	-1,435	-1,430	-0,340	-	-0,260	2,340	-1,041	0,622	-2,061	4,246	-0,497	0,247
21	2,871	3,060	3,410	2,870	2,833	2,670	2,323	3,438	2,402	5,770	3,014	9,084
22	-	-	-	-	0,003	4,430	3,471	6,590	-3,325	11,057	4,804	23,08

Table 2S. Correlation matrix in the case of experimentallly $logk_w$ values and computationally calculated indices

Correlations \(Logkw-calc.xls) Marked correlations are significant at p < .05000 N=22

	Means	Std.Dev.	CN-22°C	CN-37°C	C8-22°C	C8-37°C	C16-22°C	C16-37°C	PFP-22°C	PFP-37°C	RP18-22°C	RP18-37°C	CLogP	NCNHET	ALogP98	XLogP2	MLOGP	MLOGP2	ALOGP	ALOGP2	Average
CN-22°C	0.465	0.402	1.000	0.828	0.949	0.932	0.941	0.970	0.312	0.696	0.469	0.774	0.800	0.119	0.525	0.464	0.620	0.224	0.426	0.308	0.773
CN-37°C	0.351	0.309		1.000	0.710	0.737	0.764	0.769	0.114	0.539	0.618	0.675	0.662	0.007	0.533	0.203	0.592	0.023	0.405	0.263	0.617
C8-22°C	0.968	1.058			1.000	0.983	0.930	0.945	0.253	0.753	0.404	0.726	0.711	0.029	0.362	0.391	0.588	0.169	0.247	0.131	0.651
C8-37°C	1.114	1.012				1.000	0.920	0.930	0.235	0.809	0.450	0.736	0.662	0.042	0.306	0.364	0.535	0.170	0.184	0.088	0.621
C16-22°C	0.518	0.919					1.000	0.969	0.328	0.691	0.469	0.768	0.689	0.149	0.432	0.390	0.514	0.312	0.328	0.276	0.737
C16-37°C	0.140	0.727						1.000	0.345	0.707	0.489	0.786	0.703	0.159	0.446	0.418	0.505	0.317	0.335	0.291	0.754
PFP-22°C	2.597	1.464							1.000	-0.042	0.072	0.747	0.178	0.918	0.459	0.873	-0.260	0.754	0.562	0.855	0.783
PFP-37°C	1.675	0.970								1.000	0.618	0.408	0.376	-0.092	-0.035	0.115	0.381	0.039	-0.150	-0.236	0.299
RP18-22°C	2.568	1.293									1.000	0.463	0.072	0.163	-0.005	0.055	0.067	0.164	-0.065	0.034	0.315
RP18-37°C	1.416	1.108										1.000	0.504	0.606	0.541	0.716	0.162	0.589	0.509	0.655	0.906
CLogP	1.123	1.429											1.000	-0.079	0.768	0.414	0.818	-0.052	0.675	0.340	0.665
NCNHET	2.489	0.551												1.000	0.289	0.805	-0.482	0.741	0.434	0.796	0.630
ALogP98	1.026	1.564													1.000	0.592	0.536	0.071	0.964	0.753	0.762
XLogP2	2.457	1.538														1.000	0.011	0.533	0.697	0.799	0.824
MLOGP	0.180	1.743															1.000	-0.406	0.417	-0.034	0.304
MLOGP2	2.933	3.198																1.000	0.149	0.600	0.577
ALOGP	1.543	1.527																	1.000	0.840	0.765
ALOGP2	4.608	5.370																		1.000	0.825
Average	1.565	0.979																			1.000

Table 3S. Correlation matrix in the case of experimentallly mlogk values and computationally calculated indices

Correlations \ (mLogkw.stw) Marked correlations are significant at p < .05000 N=22 (Casewise deletion of missing data)

	Means	Std.Dev.	CN-22°C	CN-37°C	C8-22°C	C8-37°C	C16-22°C	C16-37°C	PFP-22°C	PFP-37°C	RP18-22°C	RP18-37°C	CLogP	NCNHET	ALogP98	XLogP2	MLOGP	MLOGP2	ALOGP	ALOGP2	Average
**CN-22°C**	-0.640	0.698	1.000	0.896	0.829	0.853	0.467	0.726	0.858	0.808	0.877	0.342	0.683	0.196	0.460	0.543	0.442	0.374	0.387	0.352	0.715
**CN-37°C**	-0.208	0.291		1.000	0.871	0.754	0.378	0.803	0.942	0.880	0.785	0.239	0.677	0.158	0.439	0.541	0.513	0.302	0.372	0.296	0.674
**C8-22°C**	-0.360	0.232			1.000	0.818	0.651	0.752	0.889	0.981	0.864	0.541	0.707	0.232	0.517	0.485	0.484	0.371	0.417	0.391	0.740
**C8-37°C**	-0.126	0.536				1.000	0.659	0.678	0.785	0.782	0.917	0.568	0.592	0.185	0.428	0.425	0.423	0.243	0.300	0.260	0.611
**C16-22°C**	0.009	0.143					1.000	0.376	0.467	0.608	0.688	0.976	0.578	0.112	0.576	0.258	0.487	0.060	0.464	0.381	0.568
**C16-37°C**	-0.541	0.557						1.000	0.944	0.852	0.837	0.358	0.643	-0.332	0.128	0.078	0.679	-0.040	-0.010	-0.219	0.273
**PFP-22°C**	-0.107	0.377							1.000	0.938	0.887	0.384	0.714	-0.042	0.328	0.366	0.617	0.149	0.221	0.079	0.534
**PFP-37°C**	-0.502	0.183								1.000	0.878	0.523	0.723	0.070	0.436	0.371	0.569	0.255	0.321	0.235	0.634
**RP18-22°C**	-0.392	0.646									1.000	0.632	0.692	-0.010	0.366	0.307	0.555	0.143	0.233	0.126	0.545
**RP18-37°C**	-0.045	0.158										1.000	0.525	-0.053	0.469	0.075	0.497	-0.079	0.337	0.213	0.399
**CLogP**	1.123	1.429											1.000	-0.079	0.768	0.414	0.818	-0.052	0.675	0.340	0.687
**NCNHET**	2.489	0.551												1.000	0.289	0.805	-0.482	0.741	0.434	0.796	0.609
**ALogP98**	1.026	1.564													1.000	0.592	0.536	0.071	0.964	0.753	0.827
**XLogP2**	2.457	1.538														1.000	0.011	0.533	0.697	0.799	0.832
**MLOGP**	0.180	1.743															1.000	-0.406	0.417	-0.034	0.326
**MLOGP2**	2.933	3.198																1.000	0.149	0.600	0.553
**ALOGP**	1.543	1.527																	1.000	0.840	0.841
**ALOGP2**	4.608	5.370																		1.000	0.872
**Average**	0.747	0.798																			1.000

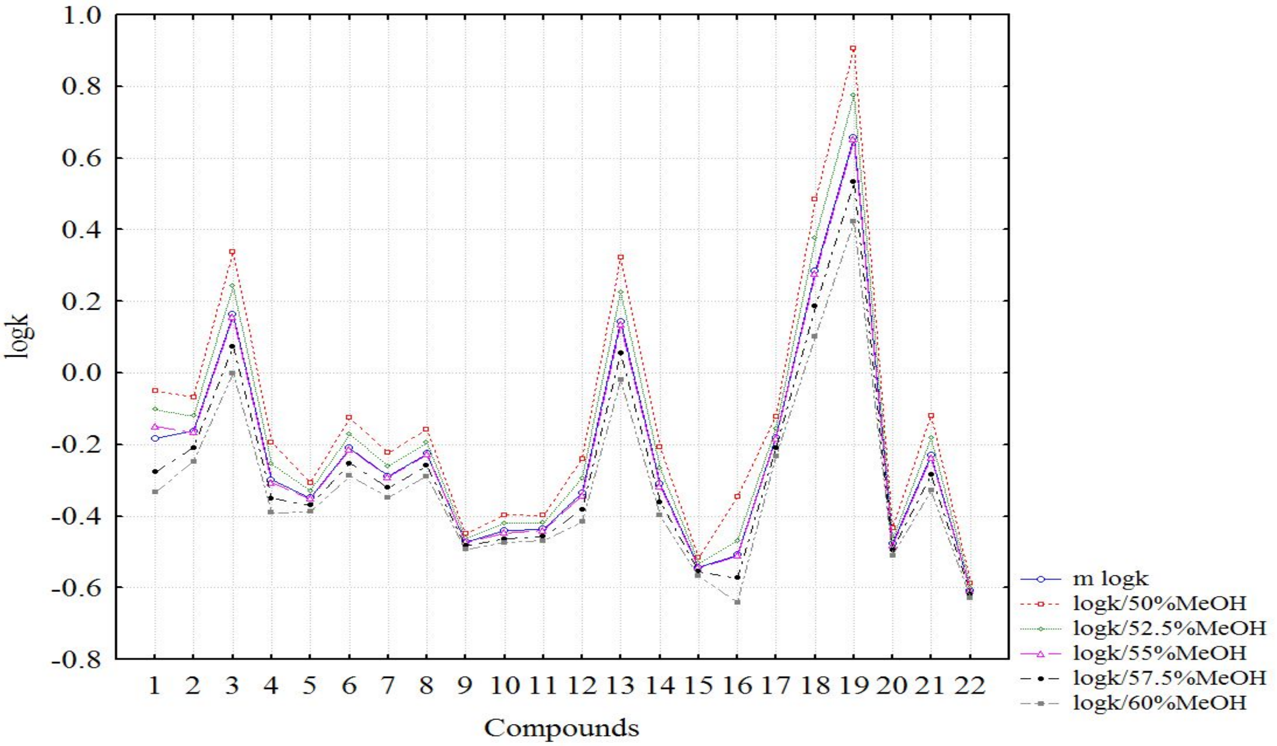

1.0
0.8
0.6
0.4
0.2
0.0
-0.2
-0.4
-0.6
-0.8
logk
1 2 3 4 5 6 7 8 9 10 11 12 13 14 15 16 17 18 19 20 21 22
Compounds
m logk
logk/50%MeOH
logk/52.5%MeOH
logk/55%MeOH
logk/57.5%MeOH
logk/60%MeOH

(a)

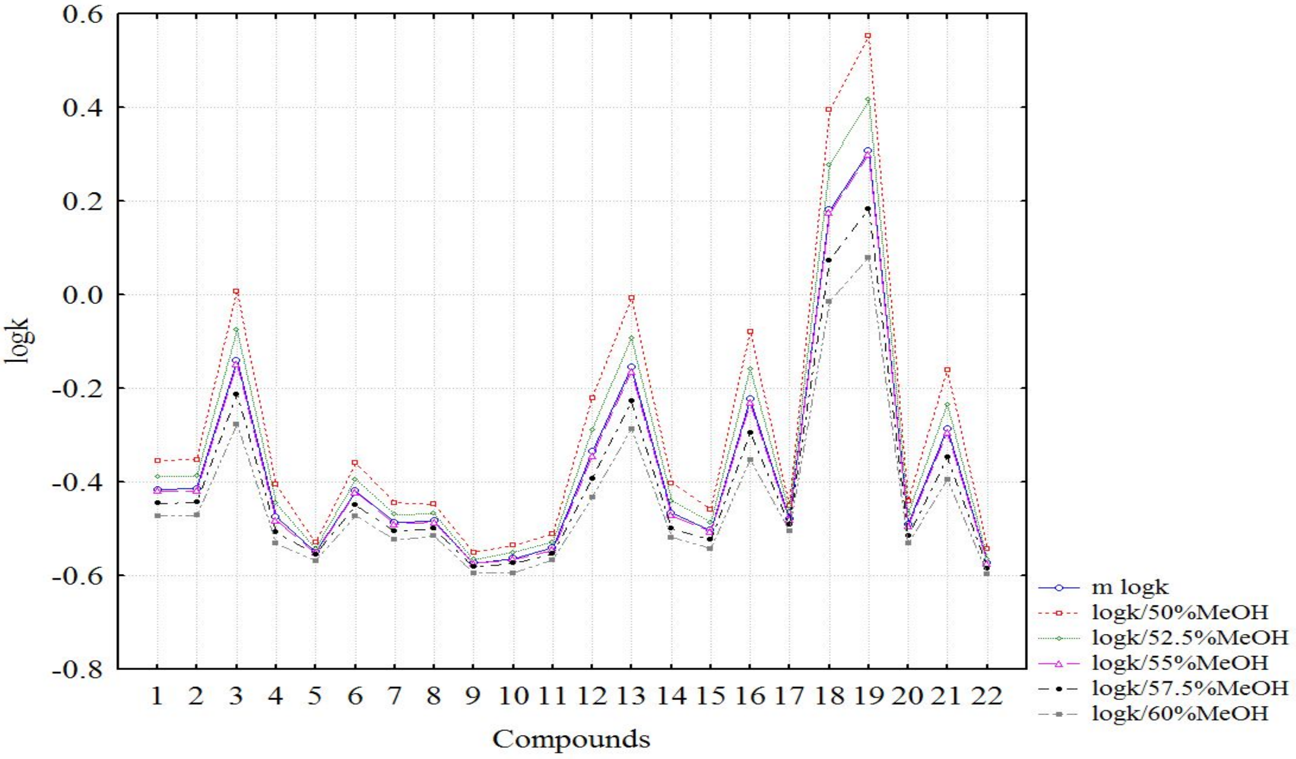

0.6
0.4
0.2
0.0
-0.2
-0.4
-0.6
-0.8
logk
1 2 3 4 5 6 7 8 9 10 11 12 13 14 15 16 17 18 19 20 21 22
Compounds
m logk
logk/50%MeOH
logk/52.5%MeOH
logk/55%MeOH
logk/57.5%MeOH
logk/60%MeOH

(b)

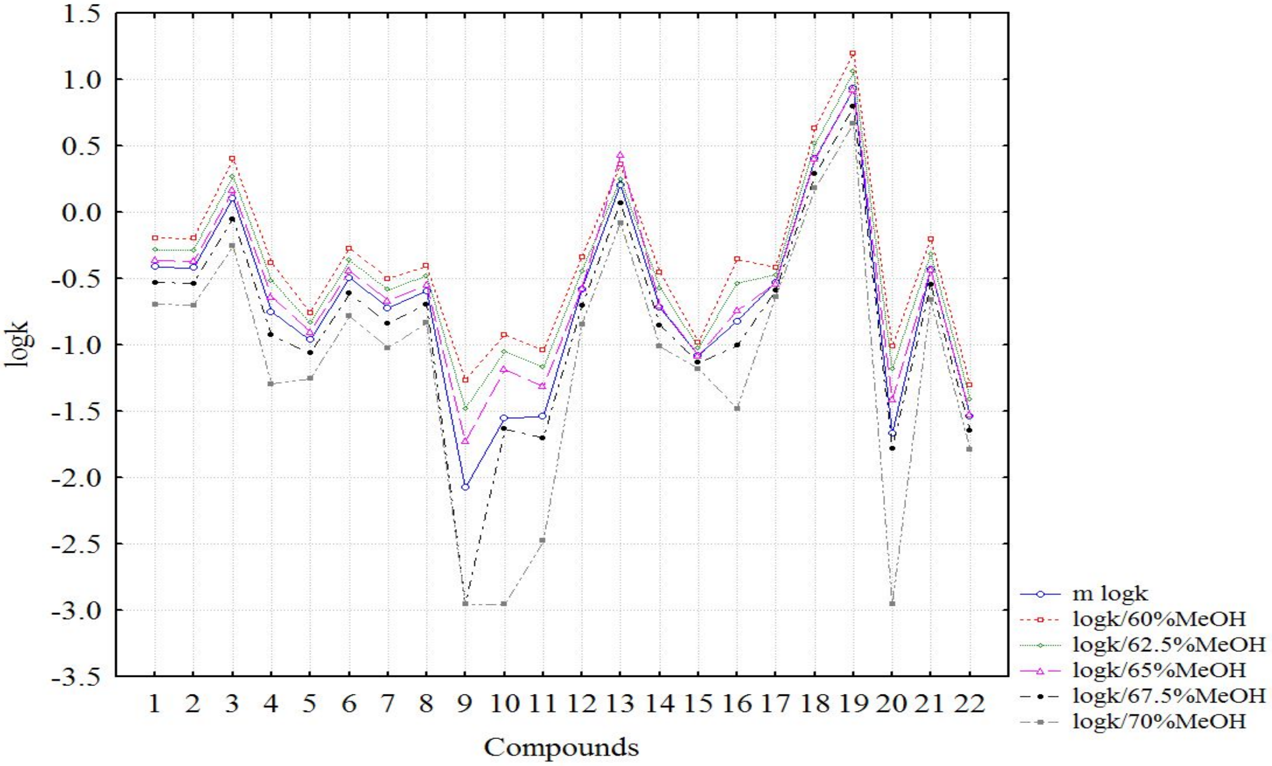

1.5
1.0
0.5
0.0
-0.5
-1.0
-1.5
-2.0
-2.5
-3.0
-3.5
logk
1 2 3 4 5 6 7 8 9 10 11 12 13 14 15 16 17 18 19 20 21 22
Compounds
m logk
logk/60%MeOH
logk/62.5%MeOH
logk/65%MeOH
logk/67.5%MeOH
logk/70%MeOH

(c)

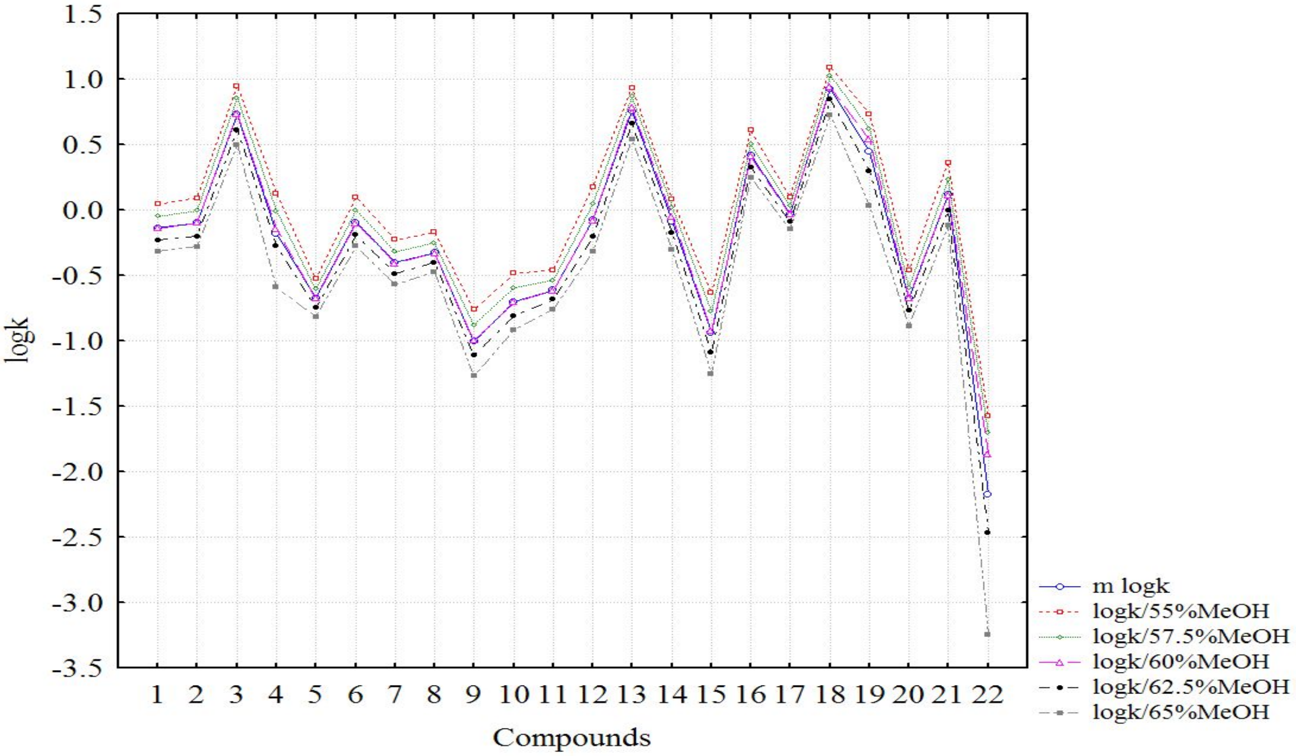

1.5
1.0
0.5
0.0
-0.5
-1.0
-1.5
-2.0
-2.5
-3.0
-3.5
logk
1 2 3 4 5 6 7 8 9 10 11 12 13 14 15 16 17 18 19 20 21 22
Compounds
m logk
logk/55%MeOH
logk/57.5%MeOH
logk/60%MeOH
logk/62.5%MeOH
logk/65%MeOH

(d)

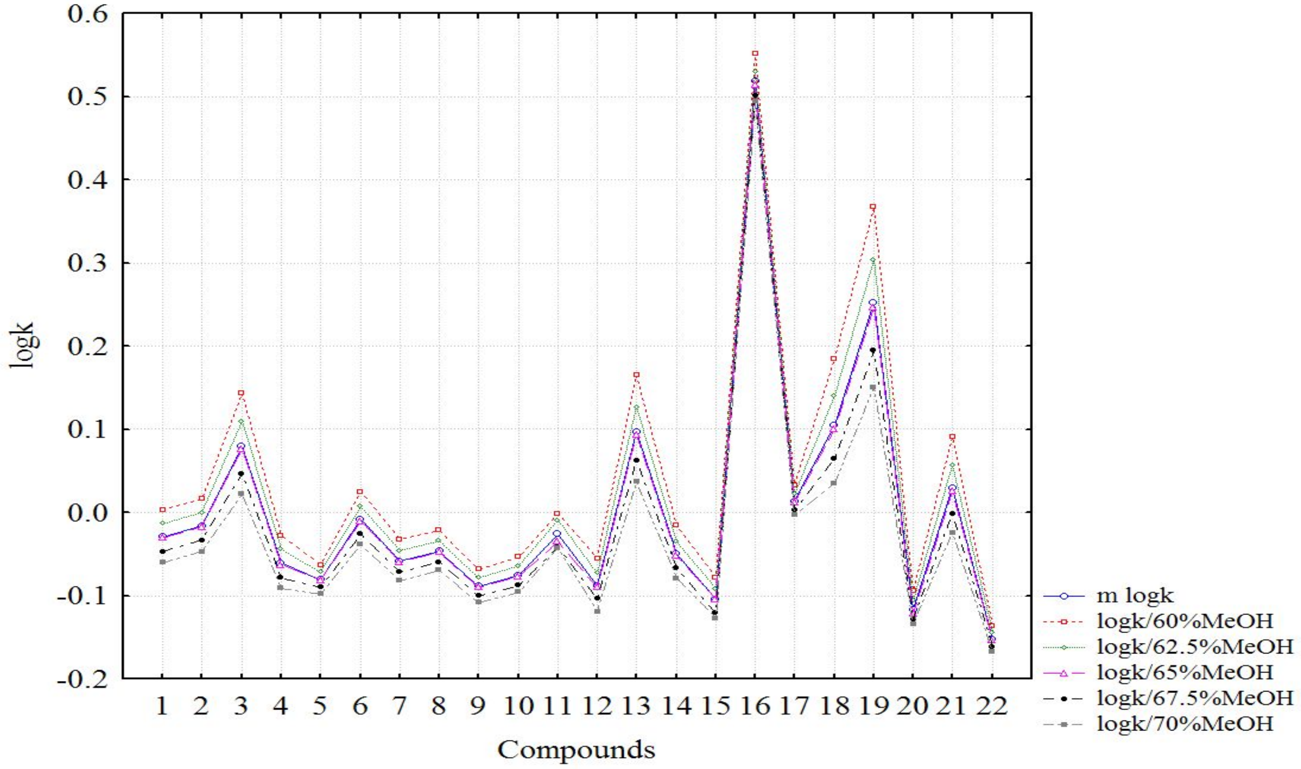

(e)

Fig. 1S Profiles of $\log k_w$ for all the investigated columns at 22 °C: (a) C8, (b) C16, (c) RP18, (d) PFP and (e) CN

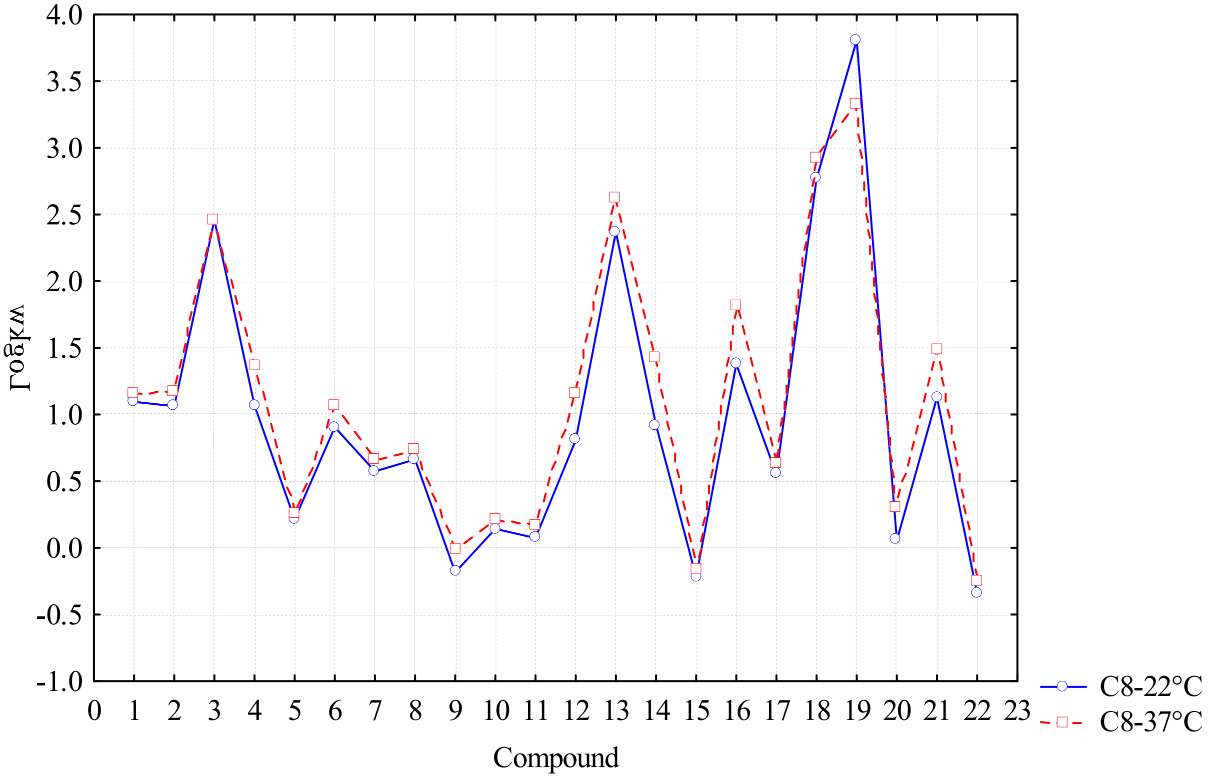

(a)

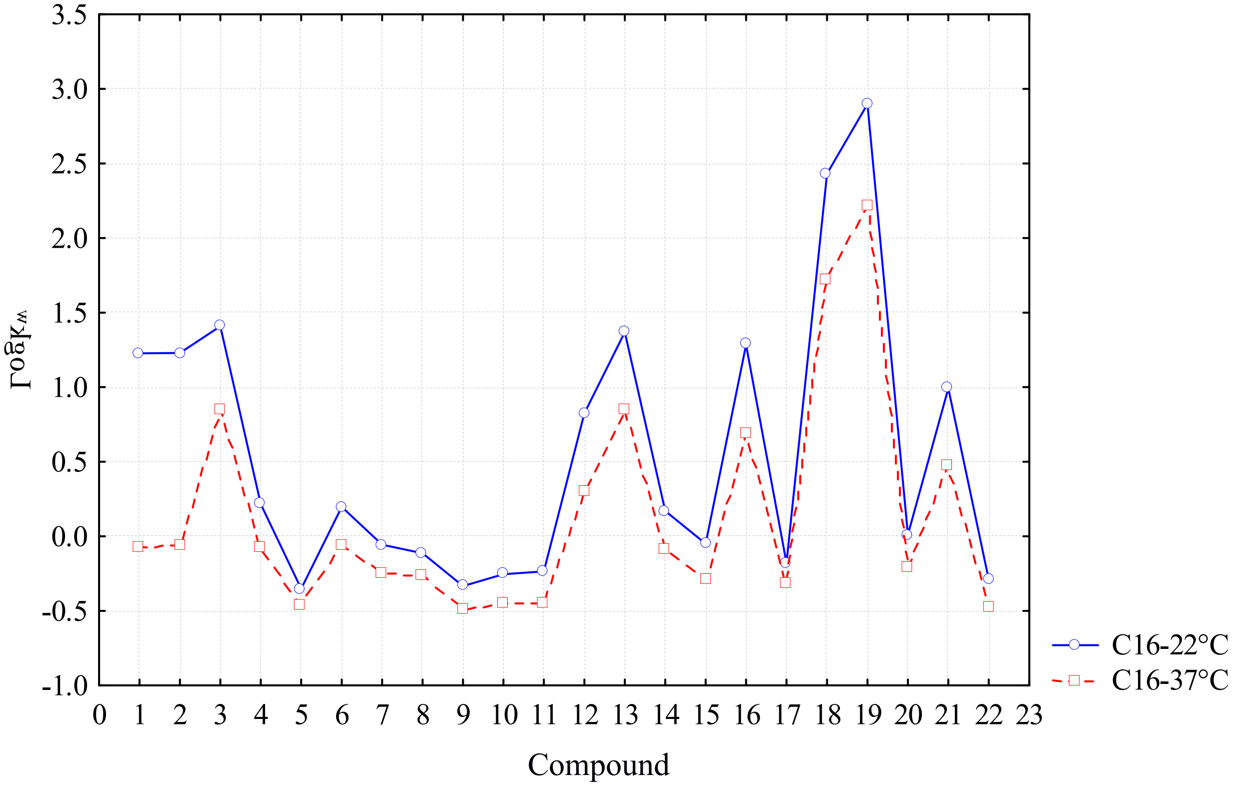

(b)

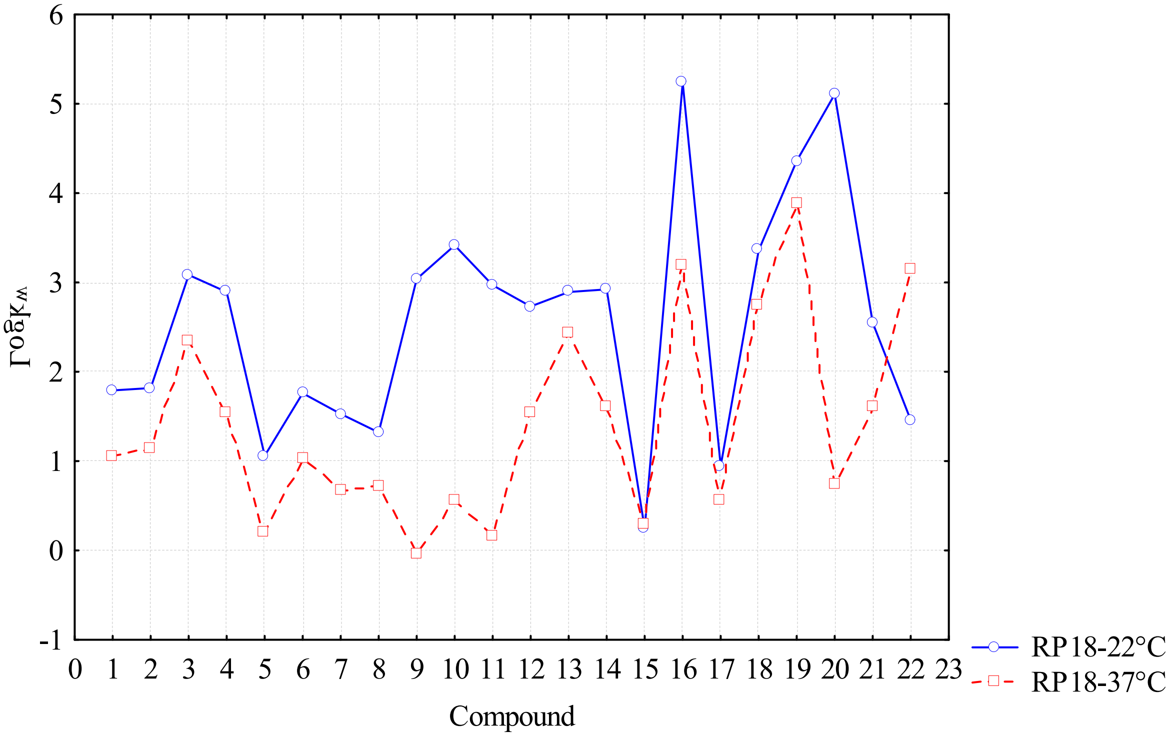

Logk$_w$
Compound
RP18-22°C
RP18-37°C

(c)

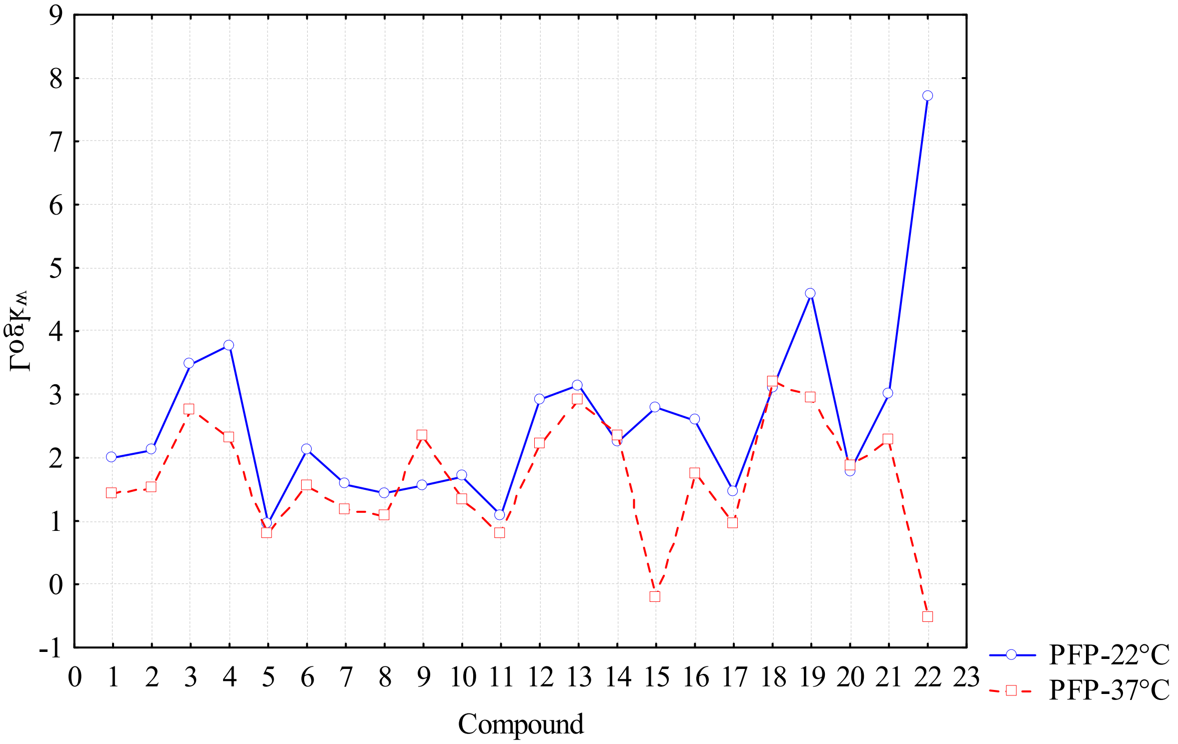

Logk$_w$
Compound
PFP-22°C
PFP-37°C

(d)

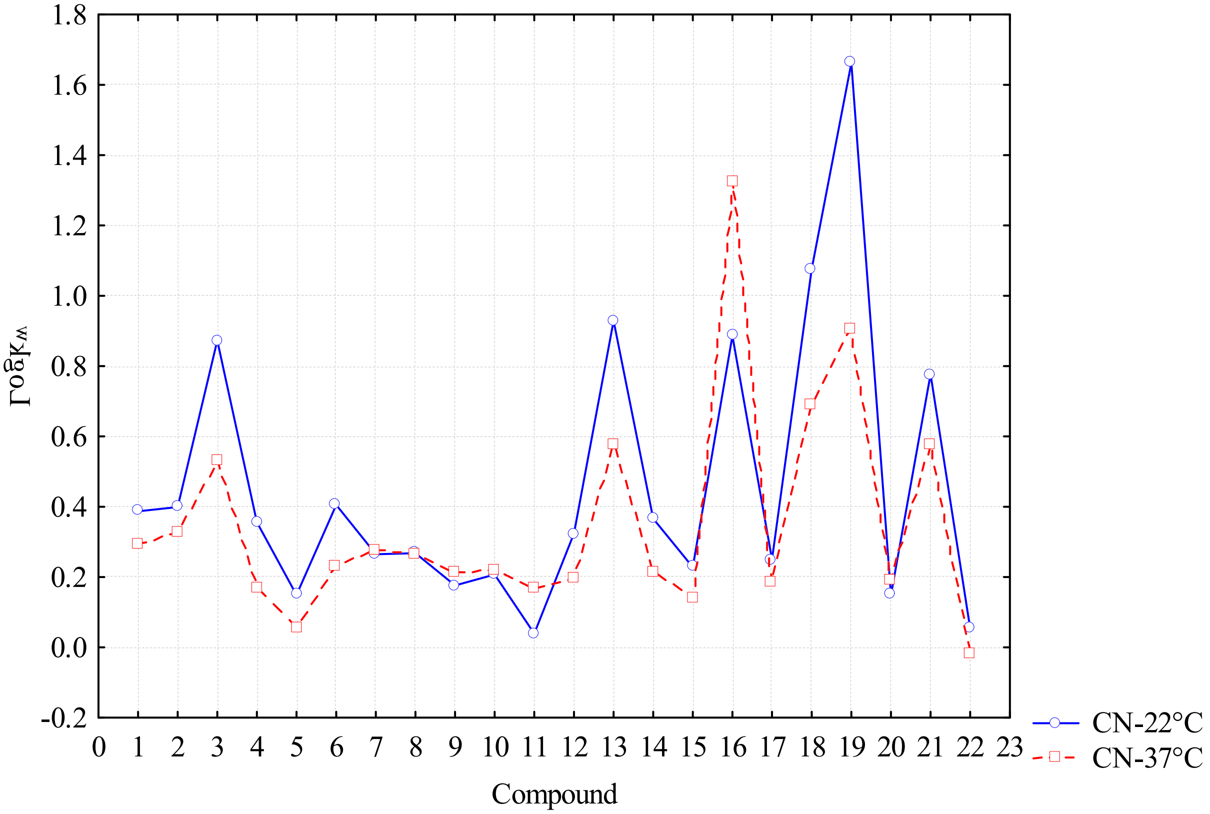

(e)

Fig. 2S Profiles of logk_w for all the investigated columns at 22 °C and 37 °C: (a) C8, (b) C16, (c) RP18, (d) PFP and (e) CN

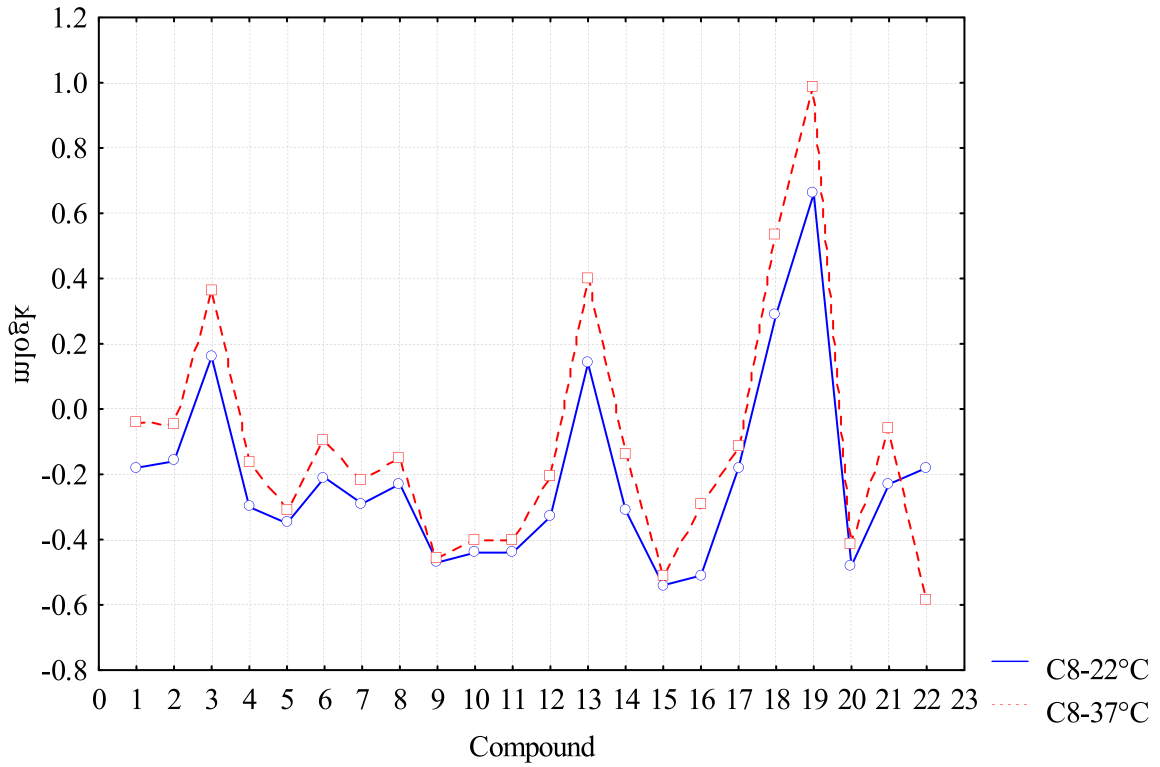

(a)

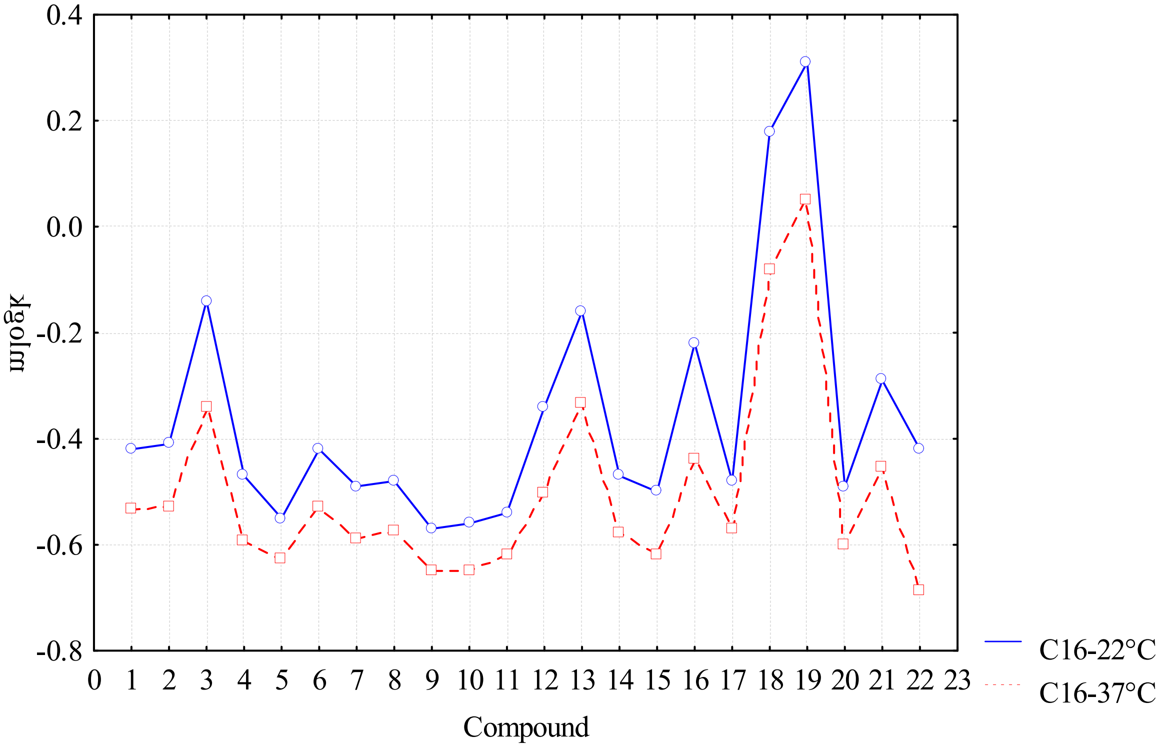

(b)

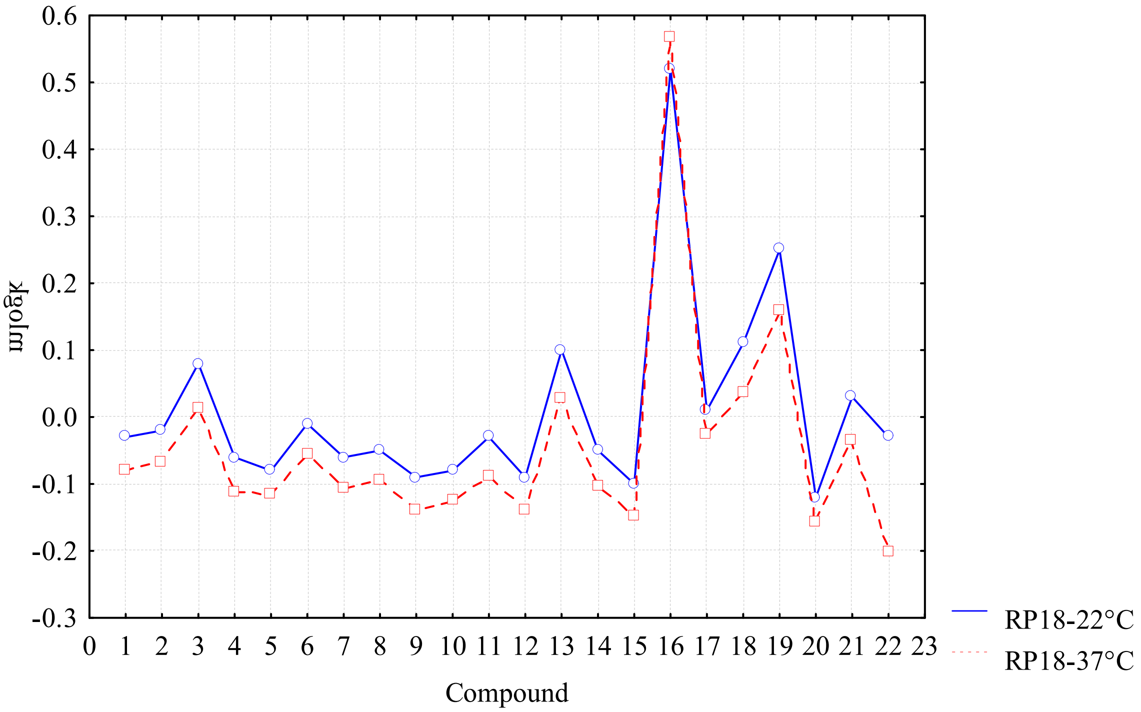

0.6
0.5
0.4
0.3
0.2
0.1
0.0
-0.1
-0.2
-0.3
mlogk
0 1 2 3 4 5 6 7 8 9 10 11 12 13 14 15 16 17 18 19 20 21 22 23
Compound
RP18-22°C
RP18-37°C

(c)

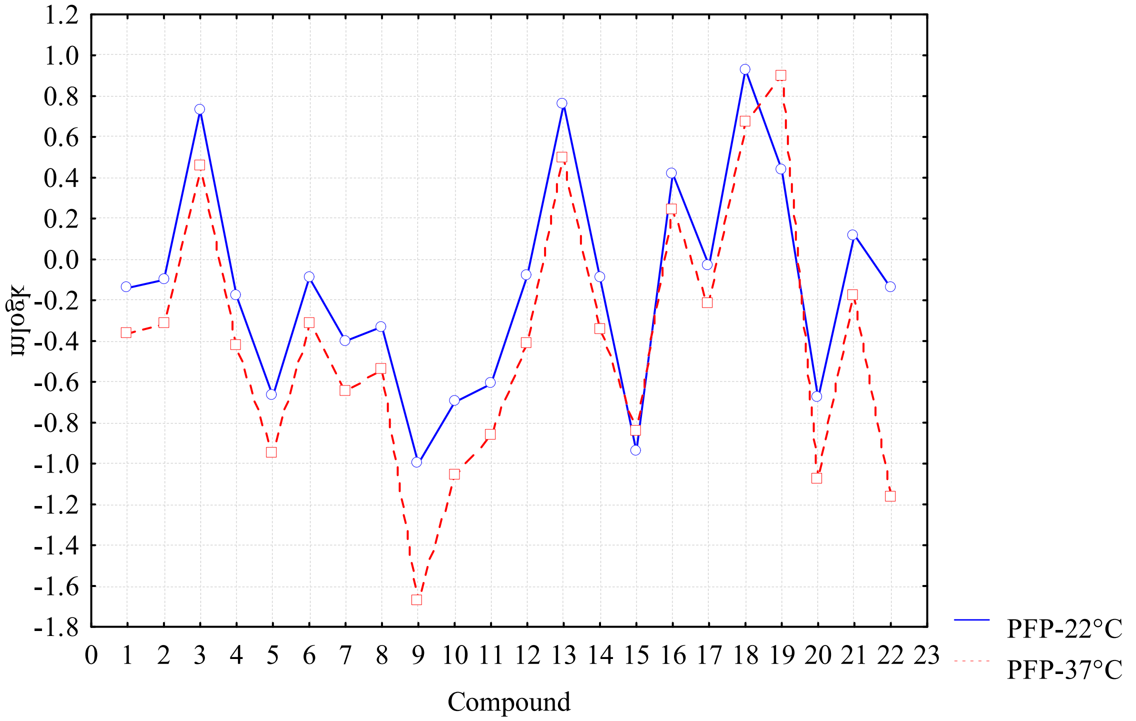

1.2
1.0
0.8
0.6
0.4
0.2
0.0
-0.2
-0.4
-0.6
-0.8
-1.0
-1.2
-1.4
-1.6
-1.8
mlogk
0 1 2 3 4 5 6 7 8 9 10 11 12 13 14 15 16 17 18 19 20 21 22 23
Compound
PFP-22°C
PFP-37°C

(d)

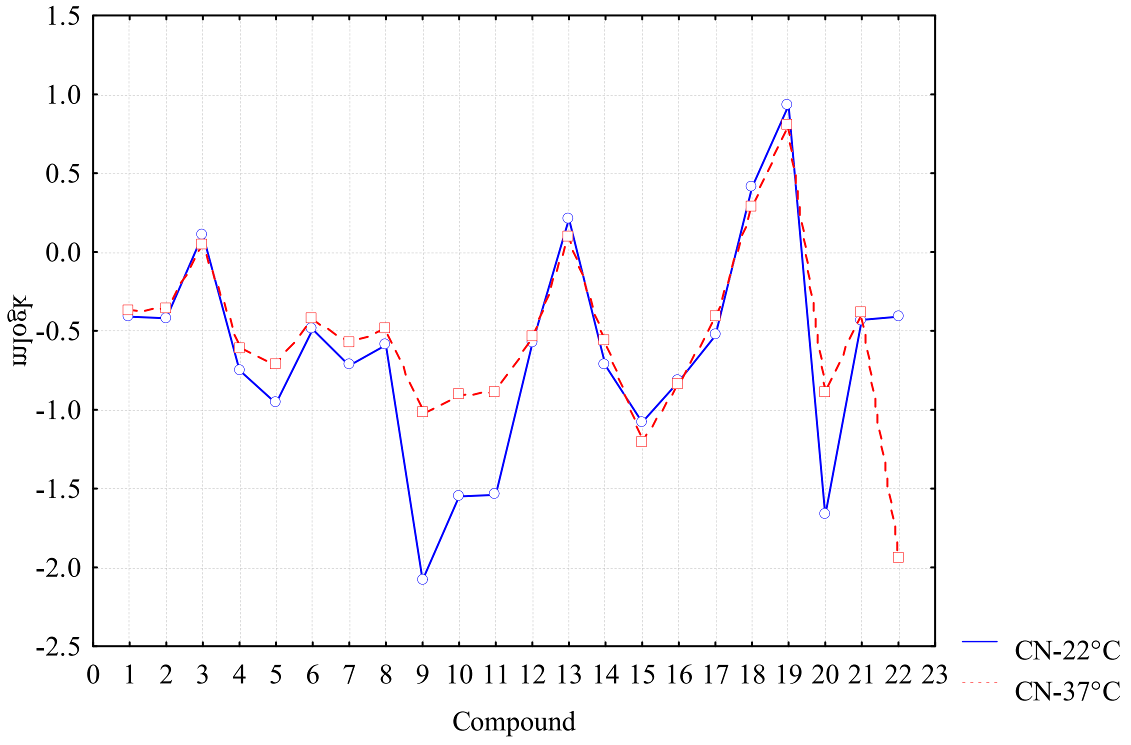

(e)

Fig. 3S Profiles of mlogk for all the investigated columns at 22 °C and 37 °C: (a) C8, (b) C16, (c) RP18, (d) PFP and (e) CN

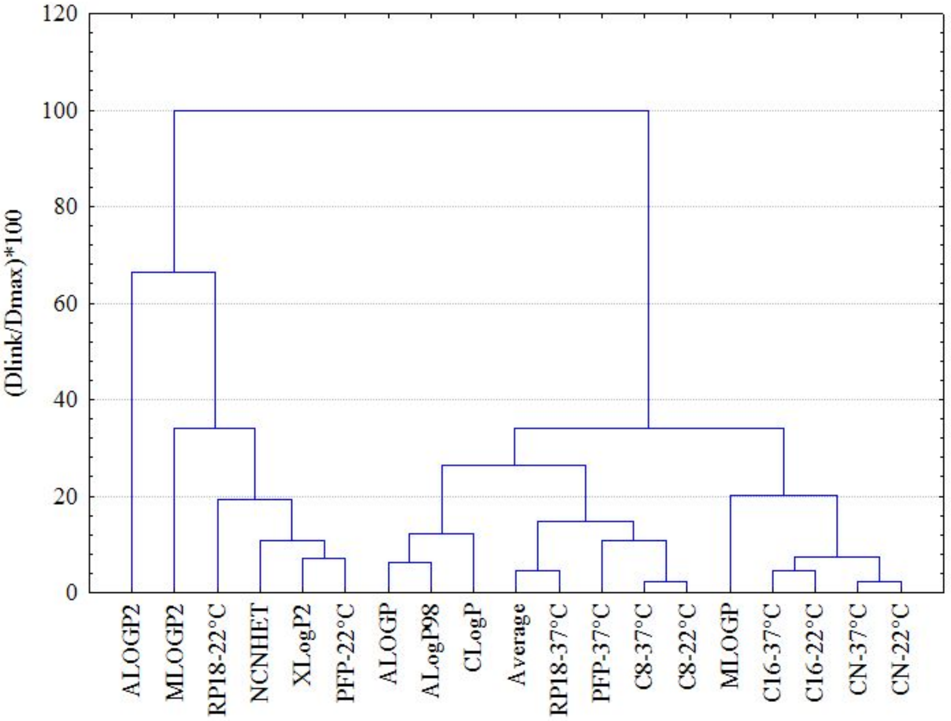

(a)

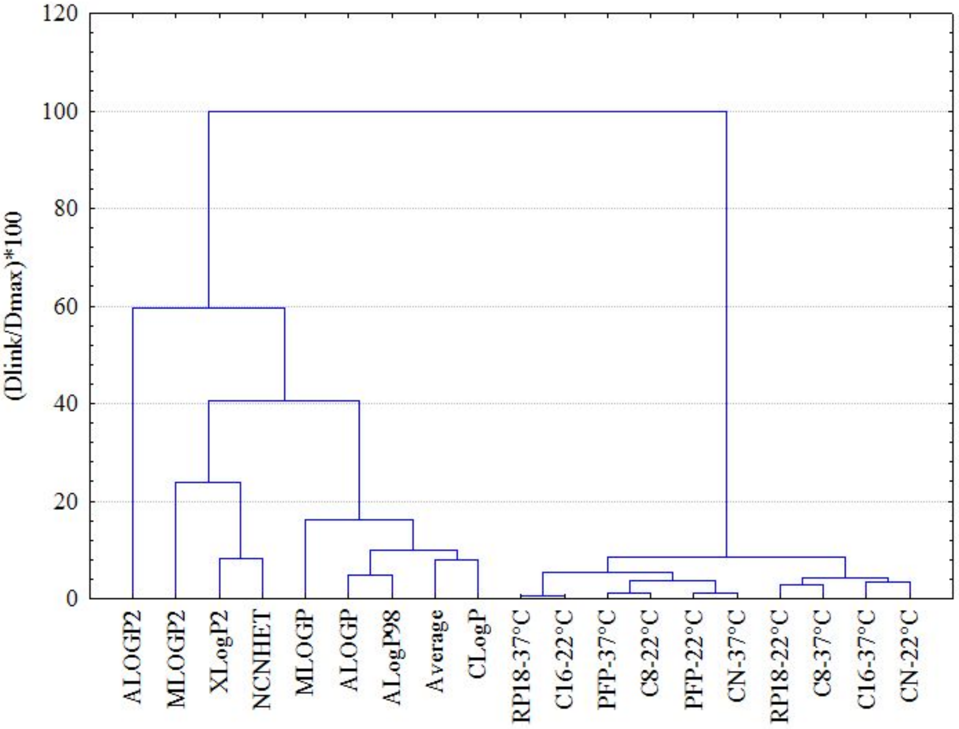

(b)

Fig. 4S Hierarchical cluster analysis dendrogram showing similarities among different chromatographic indices and computationally logP values: (a) logk$_w$, (b) mlogk

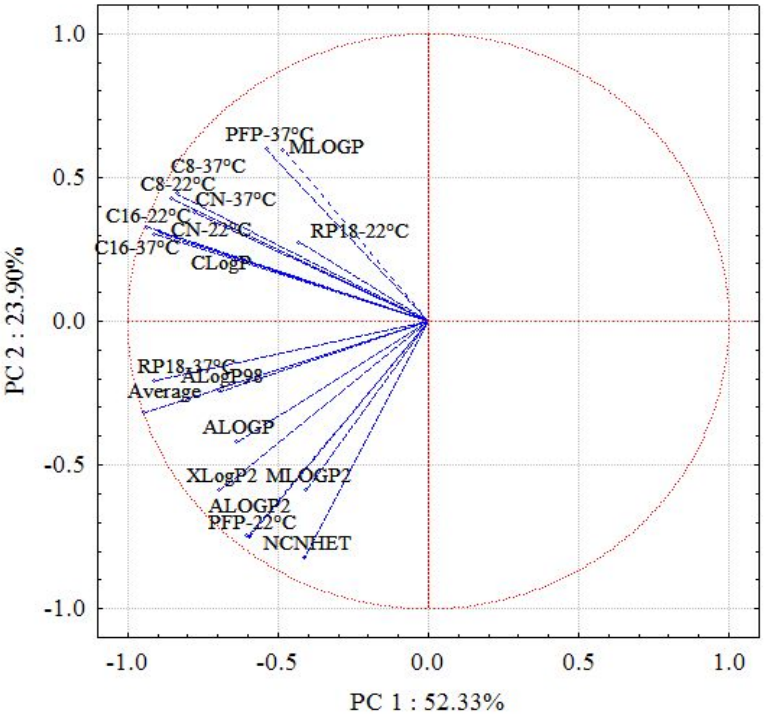

(a)

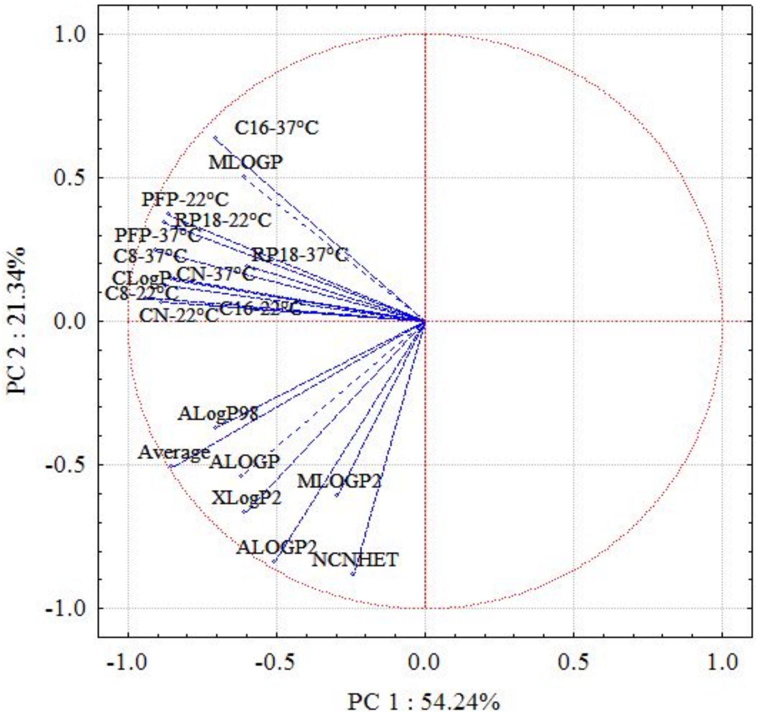

(b)

Fig. 5S Scatterplot of loadings corresponding to the first two PCs (similar lipophilicity indices are positioned close to each other): (a) logk_w, (b) mlogk

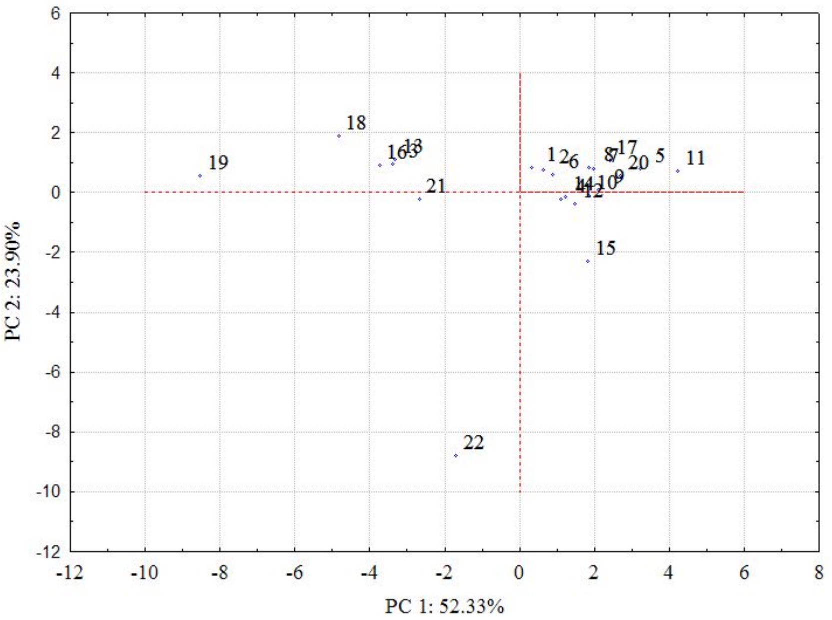

(a)

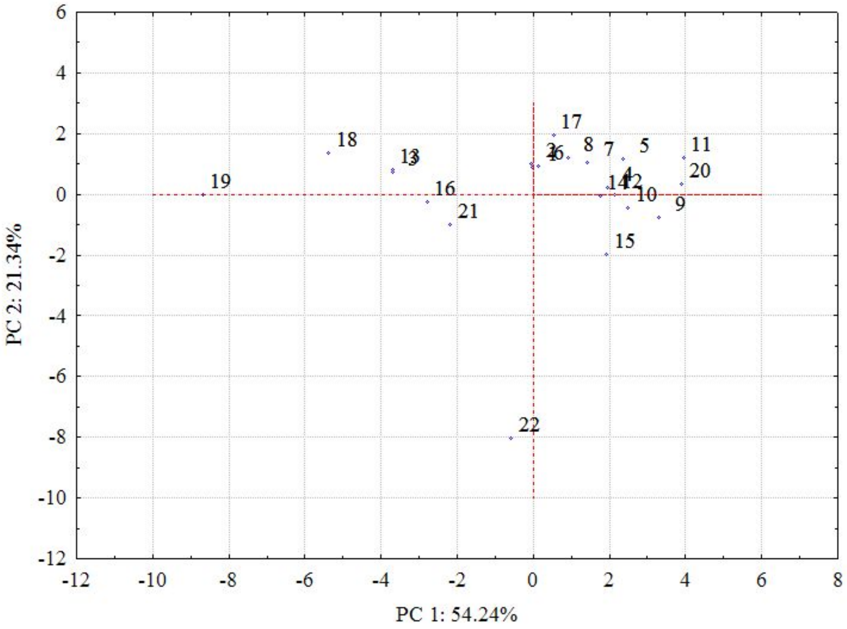

(b)

Fig. 6S Scatterplot of scores corresponding to the first two PCs (similar compounds are positioned close to each other in two distinct groups: (a) logk_w, (b) mlogk

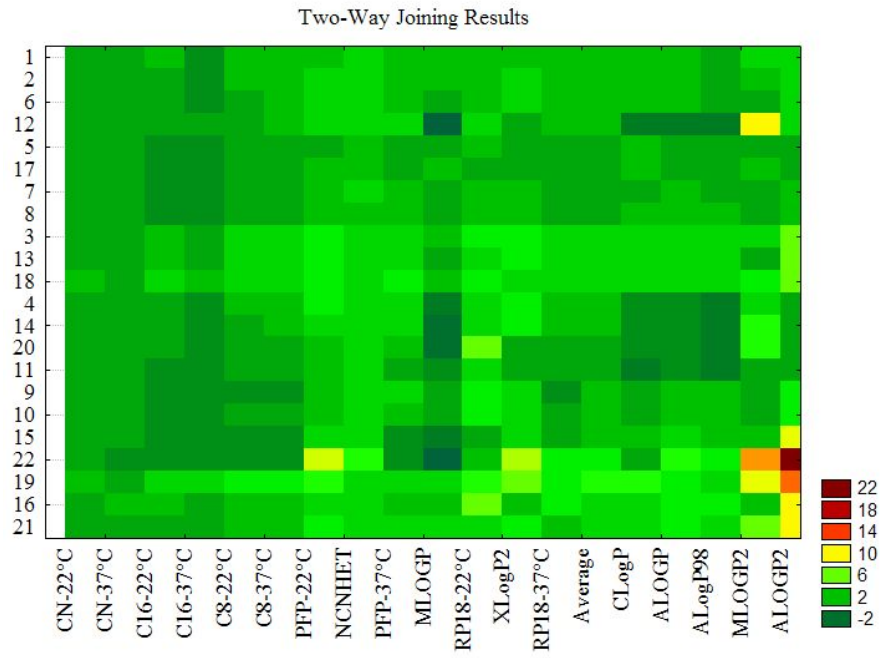

(a)

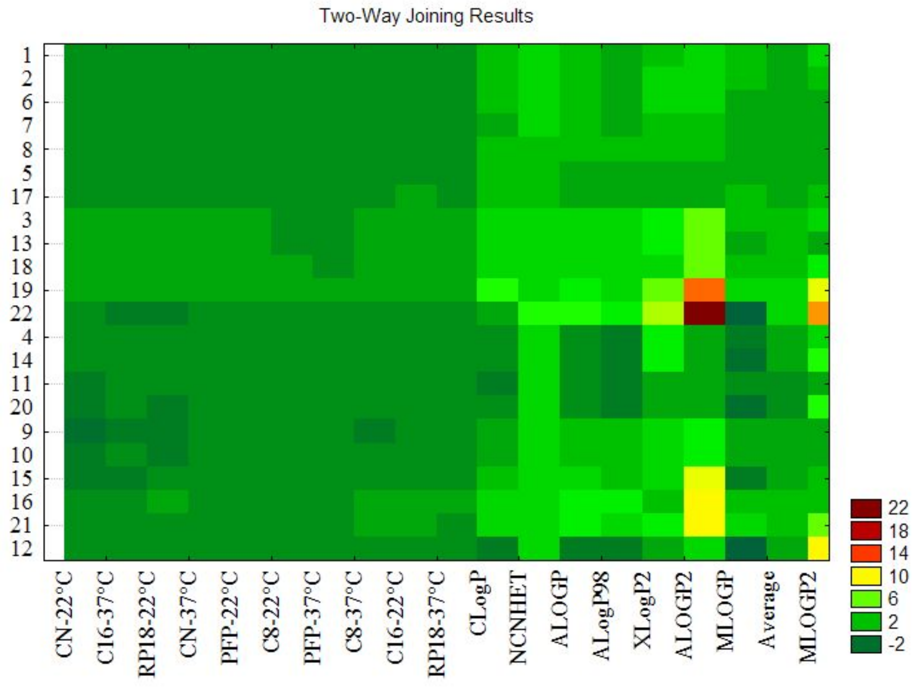

(b)

Fig. 7S Two-way joining clustering of logk$_w$ (a) and mlogk (b) including computationally logP values for all investigated columns and both temperatures
